# Acoustic Analysis of Phonation in Children With Smith–Magenis Syndrome

**DOI:** 10.3389/fnhum.2021.661392

**Published:** 2021-06-03

**Authors:** Irene Hidalgo-De la Guía, Elena Garayzábal-Heinze, Pedro Gómez-Vilda, Rafael Martínez-Olalla, Daniel Palacios-Alonso

**Affiliations:** ^1^Department of Spanish Language, Universidad Autónoma de Madrid, Madrid, Spain; ^2^Department of Linguistics, Universidad Autónoma de Madrid, Madrid, Spain; ^3^Center for Biomedical Technology, Universidad Politécnica de Madrid, Madrid, Spain; ^4^Escuela Técnica Superior de Ingeniería Informática, Universidad Rey Juan Carlos, Madrid, Spain

**Keywords:** Smith–Magenis, syndrome, speech, cepstral peak prominence, phonation stability, children

## Abstract

Complex simultaneous neuropsychophysiological mechanisms are responsible for the processing of the information to be transmitted and for the neuromotor planning of the articulatory organs involved in speech. The nature of this set of mechanisms is closely linked to the clinical state of the subject. Thus, for example, in populations with neurodevelopmental deficits, these underlying neuropsychophysiological procedures are deficient and determine their phonation. Most of these cases with neurodevelopmental deficits are due to a genetic abnormality, as is the case in the population with Smith–Magenis syndrome (SMS). SMS is associated with neurodevelopmental deficits, intellectual disability, and a cohort of characteristic phenotypic features, including voice quality, which does not seem to be in line with the gender, age, and complexion of the diagnosed subject. The phonatory profile and speech features in this syndrome are dysphonia, high f0, excess vocal muscle stiffness, fluency alterations, numerous syllabic simplifications, phoneme omissions, and unintelligibility of speech. This exploratory study investigates whether the neuromotor deficits in children with SMS adversely affect phonation as compared to typically developing children without neuromotor deficits, which has not been previously determined. The authors compare the phonatory performance of a group of children with SMS (*N* = 12) with a healthy control group of children (*N* = 12) matched in age, gender, and grouped into two age ranges. The first group ranges from 5 to 7 years old, and the second group goes from 8 to 12 years old. Group differences were determined for two forms of acoustic analysis performed on repeated recordings of the sustained vowel /a/ F1 and F2 extraction and cepstral peak prominence (CPP). It is expected that the results will enlighten the question of the underlying neuromotor aspects of phonation in SMS population. These findings could provide evidence of the susceptibility of phonation of speech to neuromotor disturbances, regardless of their origin.

## Introduction

The production of speech involves the oral coding of a message and its phonoarticulatory performance. It is a complex neurocognitive process that has been described from various cognitive approaches, ranging from the conception of the mind as software that processes linguistic information unidirectionally through rules of representation and transformation (Garrett, 1980; Fodor, 1983), to a connectionist conception that supports the existence of interconnected neuronal networks that operate in parallel in linguistic activity (McClelland et al., 1986).

Regardless of the theoretical approach taken, the neurological basis for speech production and phonation always coincides with a set of cortical and subcortical areas specialized in the organization of the message, the generation of its propositional and grammatical structure, the identification of the phonetic and phonological correlates, and the neuromotor planning of the articulatory organs involved in speech. In this linguistic activity, complex simultaneous neuropsychophysiological mechanisms occur, whose nature is closely linked to the clinical state of the individual. The complexity of neurocognitive activity occurs successfully in healthy people. In populations with pathologies of neurological origin (ictus, tumors, dementias, among others), some of the cortical areas or part of the crucial neurocognitive mechanisms in the linguistic activity are impaired. In populations with neurodevelopmental deficits, these mechanisms are also deficient and can affect phonation, speech, language, and communication, both in comprehension and production tasks.

From the phonetics, the physical sounds of the language have been studied through the characterization of the speech and the acoustic and articulatory particularities of the segmental and suprasegmental features of the languages; it has also allowed the acoustic and articulatory description of the voice production as a speech vehicle. This linguistic knowledge has a valuable application in the clinical field; thus, when speech and phonation are involved, whether for organic, functional, or neurological reasons, it is essential to know in detail the underlying characteristics and to describe the anomalies that are observed. The total of this abnormal speech profile is always a consequence of the patient’s clinical disorder.

Many studies have examined the neuromotor profile of the speech of populations with Parkinson, amyotrophic lateral sclerosis (ALS), cerebral palsy, hydrocephalus, among others, showing that speech production and phonation are compromised in the presence of diseases of neurological origin (Palacios-Alonso et al., 2020).

In the study of speech production in general and of dysarthria in particular, the starting point is the acoustic examination of speakers’ emissions. The first two formants (F1 and F2) of these vocalizations are particularly important since both are part of the acoustic correlates of articulatory activity. Formants are prominent resonances resulting from the specific configuration of the vocal tract at a given moment; specifically, F1 and F2 relate to the movement of the jaw and tongue.

In general, the first formant has an inverse relationship to the opening of the mouth: F1 is higher the lower the jaw and vice versa. The second formant has a direct relationship with the tongue: F2 is higher the further forward the tongue is in the oral tract and vice versa (Kent et al., 1999). The examination of the first two formants involves knowing the activity of some essential organs in the articulation of speech, and it is important for measuring speech intelligibility (Kent and Vorperian, 2018; Kent and Rountrey, 2020). Numerous acoustic measurements have been used in studies on the articulatory characterization of individuals with neuromotor speech problems. A classic measure used in this type of studies is the vocal space area (VSA), related to the dimensions of the acoustic vowel chart formed the first two formants of a vowel. The VSA space reflects the degree of separation between the vowels of a speaker, that is, the articulatory distinction between them in the same speaker. A reduced VSA area implies less articulatory capacity and, as a consequence, less intelligibility (Liu et al., 2005). In the ALS and Parkinson’s population, VSA is lower when compared to the normative population (Forrest and Weismer, 1997; Weismer et al., 2001; Skodda et al., 2011, 2013). A smaller area of this intervocal space has also been observed in speakers with cerebral palsy (Liu et al., 2005; DuHadway and Hustad, 2012; Levy et al., 2016). Similar results have been obtained with adult populations with Down syndrome (Bunton and Leddy, 2011), and a high degree of intrasubject variability was observed in the first two formants of the vowel /a/, but not a reduced VSA, in the X-Fragile syndrome (Zajac et al., 2006).

Another classic measure in the examination of dysarthria used as an alternative to VSA is the formant centralization ratio (FCR). This measure expresses a ratio that is extracted from the first two vowel formants, and it is expressed as: (F2u + F2a + F1i + F1u)/(F2i + F1a), where F2u is the value of the second formant of /u/, F1a is the value of the first formant of /a/, and so on (Sapir et al., 2010). Since FCR is a measure that expresses a relationship, the intravariability obtained with VSA is considerably reduced. Studies of dysarthria in patients with Parkinson’s compared to healthy populations revealed that FCR was able to differentiate between the two groups and was sensitive to the effects of treatment to improve dysarthria in patients with Parkinson’s (Sapir et al., 2007, 2010; Gómez-Vilda et al., 2017). Likewise, this same measure could differentiate the speech of Down syndrome population from healthy speakers (Moura et al., 2008).

More recent studies have proposed less conventional measures related to the kinematics of the phono-articulatory organs. One such measure is absolute kinematic velocity (AKV), which is associated with the myoelectric activity of certain facial muscles that move to the jaw, tongue, and lips (Mekyska et al., 2015; Gómez-Vilda et al., 2019a, b). The AKV has also been used to measure the articulation stability during sustained vowels emissions and allows to observe how much the first two formants fluctuate during the prolonged sustain of a vowel (usually an /a/), which leads to an analysis of the degree of articulatory position stability (Gómez-Vilda et al., 2019b).

As far as phonation is concerned, a wide variety of acoustic parameters are used to assess dysphonia. Traditionally, parameters relating to the fundamental frequency (f0) and the classical distortion parameters—jitter, shimmer, harmonic-to-noise ratio (HNR)—based on frequency and amplitude variability in consecutive glottal cycles, and on the noise-to-harmonic ratio, respectively, have been used (Lieberman, 1963; Koike, 1969; Kitajima and Gould, 1976; Yumoto et al., 1982; Hirano, 1981; Ladefoged and Antoñanzas-Barroso, 1985; Klatt and Klatt, 1990). Long-term average spectrum (LTAS) has also been used as an acoustic parameter in the analysis of dysphonia (Formby and Monsen, 1982). To assess the degree of periodicity of a voice, the cepstral peak prominence (CPP) is used as an acoustic parameter, which shows the prominence of the cepstral peak, which varies depending on the periodicity of phonation. When the voice is more periodic and richer in harmonics, the CPP is more prominent and has a greater amplitude. It was first used for phonation analysis by Noll (1964, 1967), and years later, Koike (1986) pointed out the direct relationship between the CPP and the periodicity of the voice. The automatic calculation of CPP was proposed by Hillenbrand et al. (1994) and later a variant of CPP, CPP smoothed (CPPS), was suggested (Hillenbrand and Houde, 1996). The CPPS was more useful because it had higher correlations with dysphonic voices, especially breathy voices (Hillenbrand and Houde, 1996). Over the last three decades, these cepstral parameters have been used to assess dysphonia, and a strong correlation has been found between CPP, CPPS, and the degree of dysphonia (Merk et al., 1999; Heman-Ackah et al., 2002, 2003, 2014; Halberstam, 2004; Awan et al., 2009; Maryn et al., 2009, 2010; Lowell et al., 2011; Watts and Awan, 2011; Peterson et al., 2013; Brinca et al., 2014;, among others). In fact, some authors, such as Moers et al. (2012), found higher correlations between CPP and hoarseness than between hoarseness and classical distortion parameters. Others, such as Samlan et al. (2013), observed a clear correlation between CPP and HNR but did not consider the former to be a more robust parameter in the detection of dysphonia. Currently, most researchers consider CPP and CPPS as the strongest acoustic parameters in the assessment of dysphonia severity, both in speech and sustained voice (Latoszek et al., 2018). The latter is relevant, as sustained vowels will be analyzed in this paper.

In relation to the findings on the robustness of CPP in dysarthria, it has also been shown that this parameter is sensitive to variations in phonation resulting from a neurological condition. Specifically, in research carried out with Parkinson’s patients, CPP and CPPS correlate with the degree of phonatory impairment and exhibit values below those of control groups, especially in patients with non-tremor-dominant phenotype (Burk and Watts, 2018; Cushnie-Sparrow et al., 2018; Alharbi et al., 2019; Brown and Spencer, 2020; Novotný et al., 2020). The same correlation has also been observed with phonation in patients with mild cognitive impairment (Themistocleous et al., 2020), with spasmodic dysphonia (Cannito et al., 2012), with Friedrich’s ataxia (Jannetts and Lowit, 2014; Carson et al., 2016; Vogel et al., 2017), and with cerebral palsy (van Brenk and Kuschmann, 2018; Kuschmann and van Brenk, 2019). Furthermore, cepstral parameters are significantly lower in voice analyses of populations with neurodevelopmental deficits diagnosed with Williams syndrome (Watts et al., 2008). The latter is a syndrome of genetic origin that affects the correct development of the neurological system and coincides in this sense with Smith–Magenis syndrome (SMS), the study group of this research work.

### Smith–Magenis Syndrome

Smith–Magenis syndrome (Smith et al., 1982, 1986) is a genetic disorder characterized by its low prevalence (1 in 15,000–25,000 cases), its underdiagnosis, and its chronic nature. It is caused by the haploinsufficiency of the gene retinoic acid induced (RAI1) that is due to an interstitial microdeletion of the short arm of chromosome 17 (region 17p11.2) in 90% of cases or a genetic mutation in that chromosomal region (Elsea and Girirajan, 2008; Descartes et al., 2017). SMS has a complex picture of abnormalities that include neurodevelopmental deficits (Gropman et al., 1998; Howlin and Udwin, 2002; Wolters et al., 2009), sleep and behavior disturbances (Webber, 1999; Martin et al., 2006; Wilde et al., 2013; Angriman et al., 2015; Shayota and Elsea, 2019), mild to moderate intellectual disability (IQ between 41 and 62), attention-deficit/hyperactivity disorder (ADHD) (Greenberg et al., 1996; Udwin et al., 2001; Garayzábal Heinze et al., 2011; Osório et al., 2015), and a variety of congenital problems, such as heart disease, dermatitis, hypercholesterolemia, kidney defects, nearsightedness, strabismus, recurrent otitis, scoliosis, and ear–nose–throat (ENT) disorders (Greenberg et al., 1996; Smith and Gropman, 2001; Elsea and Girirajan, 2008; Brendal et al., 2017). This complex syndrome exhibits specific physical features like short stature, obesity, brachydactyly, brachycephaly, midfacial hypoplasia, jaw prognathism, sinofrism (prominent and closed eyebrows), curved upper lip, depressed nasal bridge, among others (Smith and Gropman, 2001; Elsea and Girirajan, 2008; Gupta et al., 2016; Brendal et al., 2017).

Two of the most prominent, but least studied, features that influence the daily lives of individuals diagnosed with SMS have to do with their speech and language difficulties. Most of the individuals with SMS show language disorders that mainly affect production and are focused on the morphosyntactic, pragmatic, and phonetic–phonological levels (Garayzábal Heinze et al., 2011; Lamônica et al., 2012; Garayzábal and Lens, 2013; Wilde et al., 2016). Their phonatory profile and speech features, both children and adults, are dysphonia, high f0, excess vocal muscle stiffness, disfluencies, tachylalia, numerous syllabic simplifications, phoneme omissions, and, in general, a high unintelligibility of utterances (Hidalgo and Garayzábal, 2019; Hidalgo-de la Guía, 2019; Hidalgo-De la Guía et al., 2020).

These phonatory and articulatory particularities are very little studied but could be related to the neuromotor and behavioral profile of the syndrome. With regard to behavior, SMS individuals present serious behavioral disorders: strong tantrums, emotional lability, disruptive behaviors, hyperactivity, negativistic and defiant disorders, impulsivity, obsessive–compulsive disorders, maladaptive, motor and phonic stereotypes, polyembolocoilamania, onicotilomania, among others (Haas-Givler, 1994; Dykens and Smith, 1998; Webber, 1999; Martin et al., 2006; Wilde et al., 2013; Shayota and Elsea, 2019). Regarding their neuromotor profile, it is generally characterized by a marked delay of neurological development that explains their motor skills (see section “Neuromotor Profile of Smith–Magenis Syndrome”) (Greenberg et al., 1996; Gropman et al., 1998, 2006, 2007; Wolters et al., 2009).

### Neuromotor Profile of Smith–Magenis Syndrome

The neuromotor characteristics of SMS are the result of an altered neurological development. These neuromotor conditions of SMS patients are observed from the first year of life (Greenberg et al., 1996; Gropman et al., 2006). Classic studies with newborn children and in the first 10 years of life have outlined the neuromotor profile of these patients that includes (a) developmental delay and little weight and height gain; (b) poor interaction with the environment and low response to external stimuli; (c) low sensitivity to pain and high temperatures; (d) generalized hypotonia; (e) late onset of standing, unstable balance, abnormal tremor in extremities, poor fine and gross motor skills (severe–moderate level); and (f) late and poor babbling, oral-motor dysfunction, and speech delay (Gropman et al., 1998, 2006, 2007; Boddaert et al., 2004; Wolters et al., 2009; Maya et al., 2014). These neurological problems are also observed in the swallowing difficulties and in the hypotonia of the velopharyngeal and orofacial muscles of the patients (Sonies et al., 1997; Solomon et al., 2002). It has also been found that 75% of children with SMS have alterations in the peripheral nervous system (Greenberg et al., 1996).

The complex neuromotor deficits associated with SMS could affect multiple subsystems involved in voice and speech production, and therefore, it is important to investigate these potential effects using acoustic outcome measures. It is reasonable to assume that there are anomalies in the neurological mechanisms that interfere in phonation and speech and that the phono-articulatory organs will lack the tonicity and precision required in coarticulated speech and phonation. As seen in the previous section, several studies addressing the acoustic characteristics of speech in people with genetic syndromes (Down’s syndrome, X-Fragile syndrome) have shown abnormalities that could be due to the neuromotor deficits that occur in these disorders (Zajac et al., 2006; Moura et al., 2008; Bunton and Leddy, 2011). The voice and speech deficits associated with SMS are currently poorly understood and are important for the effective management of this disorder.

In the current study, acoustic correlates of voice and speech were analyzed as a reflection of the activity of the phonoarticulatory organs and the state of the nasopharyngeal and oropharyngeal tracts (Fant, 1960). Word articulation and speech fluency have not been considered in this exploratory study since it is the first time that a research like this is carried out in SMS, and it is convenient to limit the object of analysis. The findings would evidence the susceptibility of phonation and speech articulation to neuromotor alterations, independently of their origin.

The aim of this study is to explore whether the neuromotor deficits in children with SMS adversely affect phonation as compared to typically developing children without neuromotor deficits. These findings could provide evidence of the susceptibility of phonation of speech to neuromotor disturbances, regardless of their origin.

## Materials and Methods

### Participants

The study was carried out with an experimental group of 12 children with SMS (six boys and six girls) grouped in two age ranges: 5–7 years and 8–12 years. The sample analyzed constitutes the 17% of the total number of people with SMS in the Spanish Association of Smith–Magenis Syndrome (ASME). SMS has a very low prevalence and is underdiagnosed mainly due to the lack of knowledge that still exists about the syndrome. In Spain, the average age of diagnosis is 6.5 years (Hidalgo-de la Guía, 2019), and in ASME, there are currently 72 cases diagnosed at various ages. All participants were diagnosed by fluorescent *in situ* hybridization (FISH), which identified interstitial microdeletion in the 17p11.2 region. All children with SMS in this study come from the Spanish ASME, which has actively collaborated to facilitate our access to families and children diagnosed. The information from the experimental group can be seen in Table 1.

**TABLE 1:** Total number of normative group (NG) and Smith–Magenis syndrome (SMS) group participants distributed by gender and age.

**Ages groups**	**Group**	**Gender**	**Number of cases/IDs**
Range 1 (5–7 years)	NG	Male	3 (511O, 618O, 743O)
	SMS	Male	3 (SMS1, SMS2, SMS3)
	NG	Female	3 (517A, 612A, 637A)
	SMS	Female	3 (SMS4, SMS5, SMS6)
Range 2 (8–12 years)	NG	Male	3 (819O, 842O, 11OADS)
	SMS	Male	3 (SMS7, SMS8, SMS9)
	NG	Female	3 (10AGPC, 11AAZM, 12109A)
	SMS	Female	3 (SMS10, SMS11, SMS12)

*The number of cases and label identification is provided.*

The healthy control population sample [normative group (NG)] is a set of 12 typically developing children matched in age and sex to SMS cases. The total number of cases of NG came from the Public School “María Luisa Cañas” (Ciudad Real), where tutors and teachers were previously informed about this research. The specific exclusion criterion for constituting NG was vocal pathology, a condition provided by the speech therapist of the school and by the parents.

All the tutors and parents of the underage participants in this research signed their informed consent. This research does not violate any rights of minors and complies with all the ethical principles set out in the Declaration of Helsinki by the World Medical Association in 1964 (Povl Riis, 2003).

### Types of Samples and Recording Voice Procedure

The present study is of a purely exploratory nature since the studies addressed in SMS on the manifestations of neuromotor deficits in speech and phonation is non-existent.

In this research, samples of sustained speech were used, specifically with the vowel /a/ sustained for approximately 1 s. A sustained vowel has been chosen instead of diadochokinetic emissions or full words because it is considered the best way to contrast the initial hypothesis. To see whether the speech and voice of children with SMS reveals part of their atypical neuromotor profile, it was necessary to examine, on the one hand, the phonation under a neuromotor framework such as that of SMS—cepstral peak prominence (CPP)—and second, the stability of the articulatory position when this is held for 1 s, considering that the people articulating the vowel have neuromotor deficits. The latter is fundamental, since it relates to the behavior of the articulatory organs during sustained emission, specifically regarding the activity of the jaw and the tongue. Furthermore, that steady-state analysis without coarticulation effects surrounding phonemes is also important for generating valid measures of F1 and F2. The stable tongue and jaw position achieved in this type of speech production is optimal for phonatory analysis. It is also the most recommended way to indirectly analyze the laryngeal activity and the vocal quality.

As regards the choice of the phoneme /a/ instead of any other vowel segment, this is due to phonological issues: it is the most open vowel in Spanish and the one that favors a more natural—less forced—and more standard phonation (Vihman and de Boysson-Bardies, 1994). Several samples were taken per participant, and a total of 50 emissions were obtained. These samples were collected at different times throughout the week.

A cardioid clip-on microphone (Audio-Technica ATR-3350) was used for voice collection due to the age of the participants. In this type of study in which the voice is the object of analysis, it is essential to avoid any kind of distraction given the influence it has on phonation (Ramig and Dromey, 1996). The microphone could be a distracting element, especially in SMS population, so a small clip-on recording device was the best option. The recording device was always placed approximately 20 cm from the source (the participant’s mouth).

The files have been recorded with a sampling rate of 22,100 Hz (Smith–Magenis data set) and 48,000 Hz (normative data set), both stereo 16 bits. A small room with good acoustic conditions was the recording location for the two study groups. During the process of collecting the voice samples, only the researcher and one participant were in the recording room. To ensure that the voice samples analyzed were natural and in accordance with the phonatory characteristics of each subject, the examiner played with each participant, sang, and used the microphone for at least half an hour before collecting the voice samples. The aim was to familiarize the child with the test and the materials used.

### Acoustic Analyses Procedure

#### Preprocessing of Data

All files were filtered to subtract the mean value, to make sure no continuous bias was introduced. After that, they were all normalized in amplitude. Different sampling frequencies were used for formant an f0 calculation.

In the case of formant calculation, only the low frequencies of the spectrum were worth for authors, and a sampling frequency of 16,000 Hz was used. The reason to do so was because the first five formants lied under 8 kHz, so all the files were down-sampled to 16 kHz prior to formant calculation. Nevertheless, for f0 calculation, higher sampling frequency provided a more precise f0 calculation. Thus, in this case, a sampling frequency of 48 kHz was used. This frequency up-sampling was not going to give more precision in the case of the Smith–Magenis data set but was quite convenient, as the so calculated f0 values were easier to align with formant values if window overlap was properly selected. The resampling algorithm performs an up-sampling by an integer value “p,” followed by a low-pass filtering and a down-sampling by an integer value “q.” The relationship between p and q equals the relationship between the original and the desired frequencies (Crochiere, 1979; Crochiere and Rabiner, 1983).

#### Formant Calculation

As previously indicated, data files were down-sampled to 16 kHz. A pre-emphasis filter with a pole in 0.95 [*P*(z) = 1 − 0.95z^–1^] was applied to every file to enhance higher frequencies. An all-pole model system was considered for speech. To calculate such system, a linear predictive coding (LPC) model with covariance algorithm was calculated in block processing. For every file, LPC models of orders 12, 14, and 16 were calculated, in most cases, 12 order being the optimum compromise. Nevertheless, older children tended to show lower formants, so a higher order (14) was sometimes necessary to separate first two formants in a Spanish /a/ vowel. The window length for the block processing was 256 ms (4,096 samples). This is a very long window, but the data set consisted of sustained vowels, so slow variations in the position of the formants were expected. The window displacement was 8 ms (128 samples). That implied an overlap of 96.9% between windows.

The formants were calculated as the frequencies that corresponded to the positions of the roots of the polynomial of the LPC model. Some restrictions were taken to consider a root being a real formant: the minimum value for the radius of the first formant should be 0.84, and the minimum imaginary part for that first formant should be 0.05. Then, only frequencies above 150 Hz were considered for the first formant. Consecutive formants should be calculated from roots of radius higher than 0.82, 0.8, 0.78, 0.76, and 0.74. This less strict restriction as the order of the formant increase was due to the lower level in energy of the formants as frequency increased.

#### f0 Calculation

**FIGURE 1:**
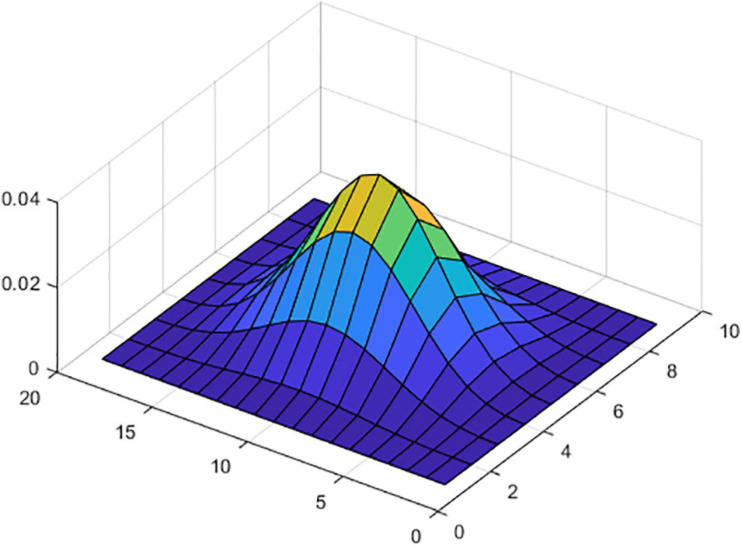
Filter coefficients.

For f0 calculation, files were resampled to 48 kHz. The value of f0 was searched between a minimum of 40 Hz and a maximum of 700 Hz. The period in samples that corresponded to these two frequencies were fs/40 (T_*max*_) and fs/700 (T_*min*_) samples. The algorithm was based in cepstrum. Files were block processed with a window length of 64 ms (3,072 samples) and a displacement of 8 ms (384 samples and 87.5% overlap). The time displacement was the same as in the case of the formant calculation. Every frame was multiplied by a Hamming window function, and the real cepstrum was calculated and put together in a matrix. The so obtained cepstrum matrix was then filtered with a 2D filter of size 19 × 9 (19 files and nine columns). This filter was obtained as the product of two matrices: the first matrix consisted of 19 files of Blackman window vectors of size 9, and the second one consisted of nine columns of Blackman window vectors of size 19. The so obtained filtering function can be viewed in Figure 1.

After that filtering, cepstrum vectors were compensated to enhance low frequencies. The period in the samples of every frame was calculated as the position of the maximum of every column in the cepstrum-filtered matrix between T_*min*_ and T_*max*_. On some occasions, the second or the third peak of the cepstrum (situated at double and three times the samples of the first one) could be more prominent than the first one, although low frequency enhancement was accomplished. Those cases were detected, and if the first peak was over the 60% of the value of the most prominent one, the value was corrected. When the value of the first peak was even lower than the 60% of the most prominent one, we assumed that a subharmonic of the f0 was present.

#### Cepstral Peak Prominence Calculation

An estimate of the goodness of the cepstral peak was calculated for every cepstrum frame. The real cepstrum of the windowed frames was smoothed with an FIR filter of 25 coefficients with a Hamming window impulse response. The cepstrum signal was then limited between T_*min*_ and T_*max*_. The maximum (cepstral peak) of the so constrained signal was calculated. Finally, the CPP was obtained as the difference between that value and the average of the rest of the cepstrum signal between T_*min*_ and T_*max*_. This value was also smoothed between consecutive frames, with a filter with a window length of 50 ms.

### Stages of the Study and Statistical Analysis

**FIGURE 2:**
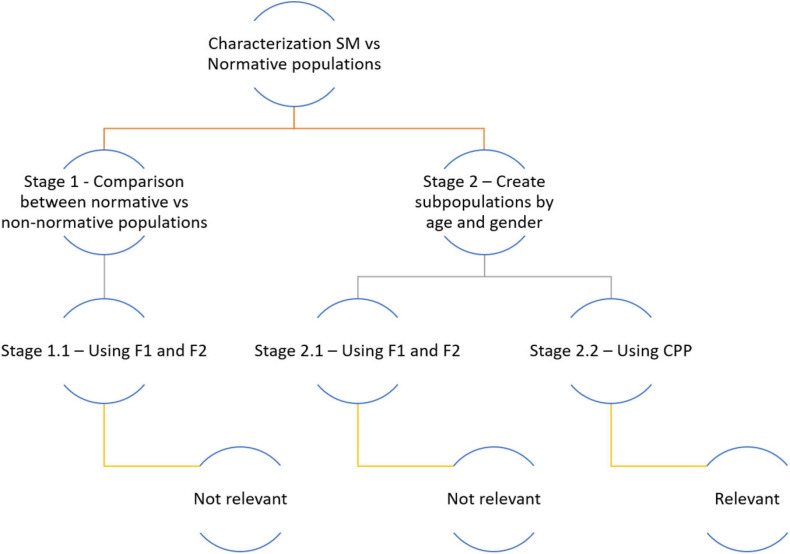
Phases of the study and statistical treatment of the data.

For the statistical analysis of the data and their comparison with the control population, parametric and non-parametric statistical tests were used. The analysis of the data was complex and required a thorough examination divided into different stages, which are summarized in Figure 2. In stage 1, a first comparison of F1 and F2 between the non-normative (SMS) and the normative (NG) group was carried out. In stage 2, due to the absence of relevant results, it was considered to carry out a second comparison between SMS and normative cases of the same age range and gender, i.e., one SMS child of rank 1 versus each of the three normative children of the same age range and gender (Table 2) and so on. In this phase, on the one hand, the results of the F1 and F2 analysis of both groups (Stage 2.1.) were compared; on the other hand, the outcomes of the phonation analysis (CPP) (Stage 2.2.) were contrasted. As can be seen in Figure 2, the relevant results were found in the latter stage.

**TABLE 2:** Summary of statistical values for cepstral peak prominence (CPP).

**Ages groups**	**Gender**	**Number of cases**	**Group**	**/n/**	**Mean (CPP)**	**SD (CPP)**
Range 1 (5–7 years)	Female	3	NG	201	0.043927185	0.005928974
		3	SMS	229	0.039863101	0.007990531
	Male	3	NG	191	0.052336316	0.004689291
		3	SMS	411	0.036588257	0.007230023
Range 2 (8–12 years)	Female	3	NG	231	0.053534125	0.004703601
		3	SMS	701	0.042122619	0.006642816
	Male	3	NG	321	0.050821868	0.005620712
		3	SMS	425	0.045624435	0.007151963

*The added columns provide information about cluster (range of age), gender, group (NG/SMS), the number of samples, the mean, and the standard deviation of CPP.*

**FIGURE 3:**
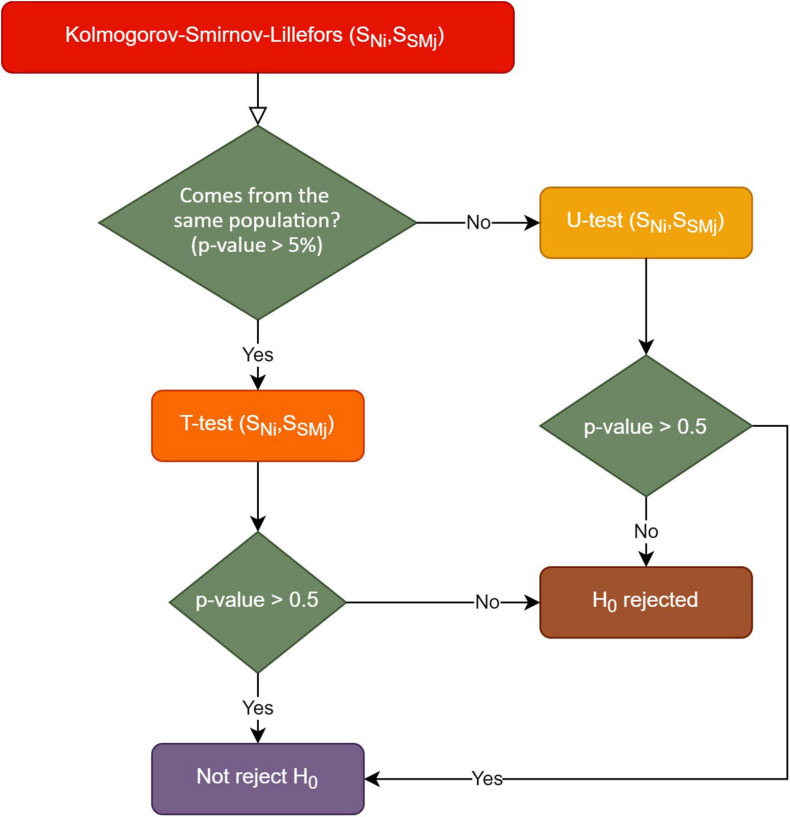
Statistical analysis according to the test rules used.

The approach followed in the statistical analysis was conditioned by the statistical restrictions set by each test used (Figure 3).

Given the two distributions, the first step was to check if these distributions followed or not a normal trend. Due to the two-stage analysis process in this study, the overall distributions and age/gender subgroup distributions were tested for normality. The Shapiro–Wilk’s test is used when the number of samples is <50, whereas the Kolmogorov–Smirnov test is used when samples are 50 or more. Due to the number of samples per participant (| *N*| ≥ 50), the Kolmogorov–Smirnov (KS) test was appropriate. When distributions were non-normal as evidenced by a KS test *p* < 0.05, the Mann–Whitney, or *U* test, was used to determine group differences.

## Results

The outcomes of the acoustic analysis are the result of a complex statistical examination of the data. It was in Stage 2.2 (Figure 2) that relevant results were obtained in terms of the values of the statistical tests used (Figure 3). Two data analysis approaches were performed. The first approach involved individual comparisons for each age and gender matched SMS and NG participant pair, using the 50 repeated samples for each participant. The second approach involved the analysis of four collective clusters—range 1 and 2 male and range 1 and 2 female, respectively. In both analysis approaches, the acoustic analysis of phonation was based on CPP extraction. The results of the statistical tests indicated that there were significant differences between phonation of SMS and NG groups. Likewise, in Table 2, the summary of statistical values for CPP such as cardinality, mean, and standard deviation were provided divided into range of age, gender, and group (NG and SMS).

At this point, in the first analysis approach, the outcomes of the acoustic analysis of the phonation of SMS cases were compared with the age- and gender-matched NG individuals. The results of the *T*- and *U*-test are shown in Table 3. In yellow color, *T*-tests with not significant *p* value are depicted (two cases out of 36); in red color, Mann–Whitney–Wilcoxon tests (*U*-test) with not significant *p* values are illustrated (three out of 36). Finally, each cell without background represents significant *p* values, using *U*-test (31 cases out of 36).

**TABLE 3:**
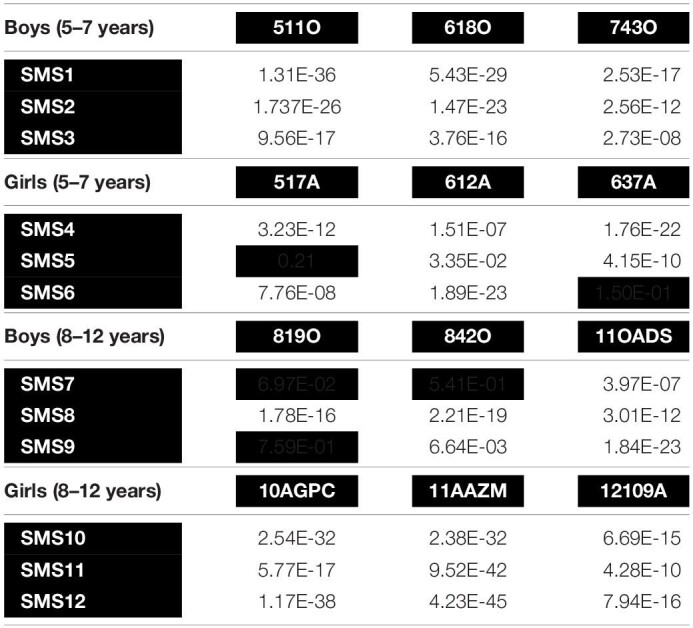
values comparison of cepstral peak prominence (CPP) with T- and *U*-tests*.

*In yellow: *T*-test not significant. In red: Wilcoxon (*U*-test) not significant. Without background: (*U*-test) significant.

The table above shows the results of the T- and *U*-tests comparisons of the CPP data extracted from the acoustic analysis of /a/. The aquamarine rows show SMS group cases, and the red pale columns show the NG cases. As can be seen, the results have been grouped by age range and gender. All results rejected the null hypothesis (*p* < 0.05), except for the boxes highlighted in red and yellow. That is, the non-highlighted results reflect that the CPP values of SMS and NG cases are significantly different, which means that these participants have distinctly different phonations.

**FIGURE 4:**
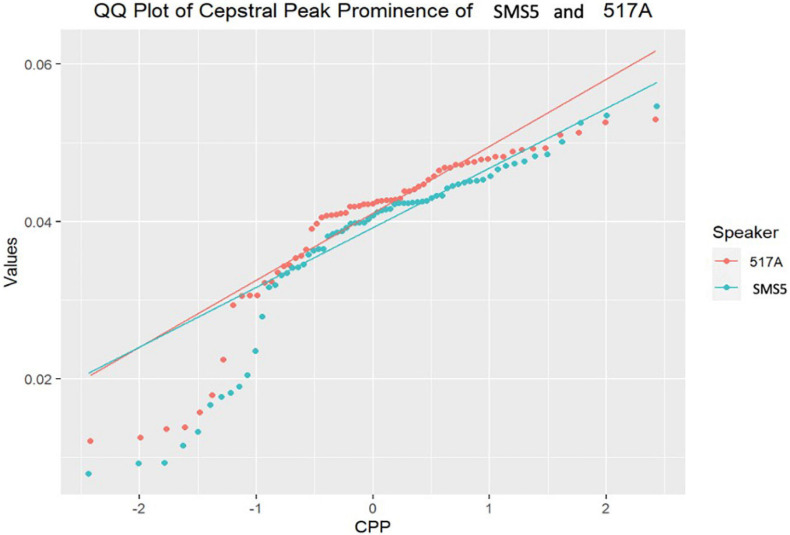
Q–Q plot of SMS5 and 517A cepstral peak prominence (CPP) distributions.

**FIGURE 5:**
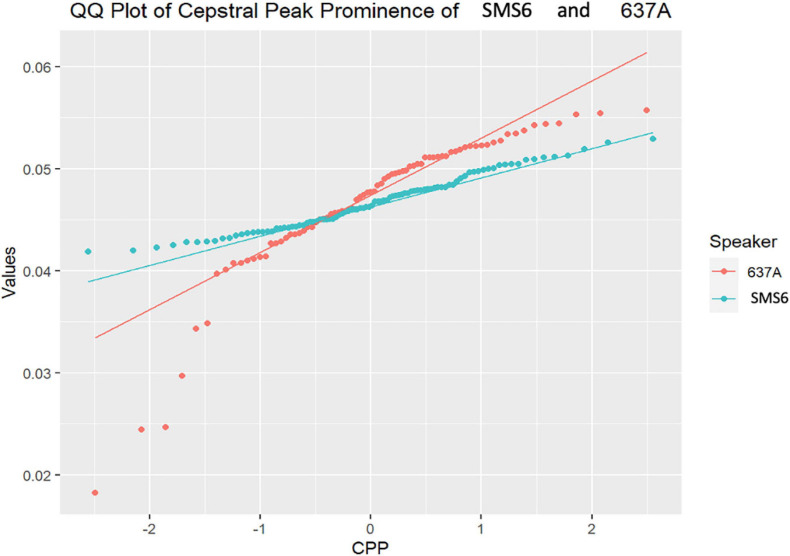
Q–Q plot of SMS6 and 637A cepstral peak prominence (CPP) distributions.

**FIGURE 6:**
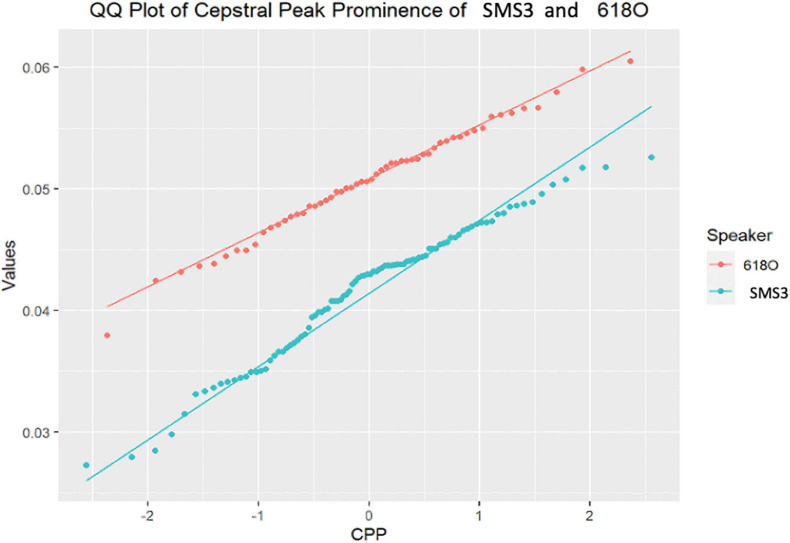
Q–Q plot of SMS3 and 618O cepstral peak prominence (CPP) distributions.

**FIGURE 7:**
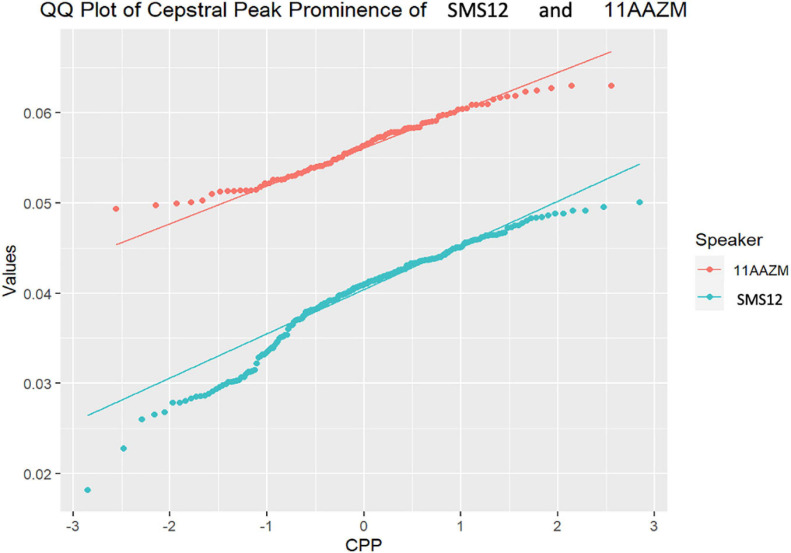
Q–Q plot of SMS12 and 11AAZM cepstral peak prominence (CPP) distributions.

As follows, in Figures 4–7, the three-representative kind of outcomes in Table 3 are shown; using Q–Q plots reflecting the sample distributions, where it can be seen that SMS5 vs. 517A (in yellow) and SMS6 vs. 637A (in red) cases are above the *p* value. Figure 4 is a Q–Q plot of SMS5 and 517A CPP distributions.

Figure 4 above illustrates that the distributions of SMS5 and 517A have similar trends (non-significant *T*-test, *p* = 0.21), with similar distributions that intersect.

In Figure 5, the Q–Q plot between SMS6 and 637A is depicted. As in Figure 4, the two case distributions intersect and do not show statistically significant differences (*p* = 0.15). However, in this case, these distributions do not follow a normal trend, and therefore, the non-parametric *U*-test was used.

In contrast to the previous cases that were not significantly different, the majority of case comparisons were significantly different. For example, Figure 6 depicts the Q–Q for the comparison of SMS3 and 618O, with distributions that were widely separated and significantly different as tested with the *U*-test (*p* = 3.76E−16).

Likewise, in Figure 7, the distributions for the comparison of SMS12 and 11AAZM are completely distinct, with statistically significant differences (*p* = 4.23E−45).

As can be shown in the aforementioned figures, Q–Q plots provide easy-to-read behavior in participants. For instance, if populations cannot be distinguished, or put in other words, belong to the same distribution, the set of points are located very close (see Figures 4, 5 respectively). However, if the statistical test returns a *p* < 0.05, there exists statistical significance between non-normative and NGs (see Figures 6, 7 respectively). Therefore, these plots depict two perfectly separated distributions.

Concerning the second study, the process described in Figure 3 was carried out for each cluster—old girls/boys and young girls/boys—respectively. In all cases, Kolmogorov–Smirnov test rejected the null hypothesis; in other words, both contrasted distributions did not follow a normal trend. For this reason, the non-parametric *U*-test was the inferential statistic used to determine if there was a significant difference between these two populations (NG and SMS). As shown in Table 4, *p* values were well below 0.05.

**TABLE 4:** values the comparison of cepstral peak prominence (CPP) with *U*-test.

**Group**	**Gender**	**Population**	***p* value**
Range 1 (5–7 years)	Female	NG	1.89E−06
		SMS	
	Male	NG	4.84E−79
		SMS	
Range 2 (8–12 years)	Female	NG	3.62E−84
		SMS	
	Male	NG	7.02E−19
		SMS	

**FIGURE 8:**
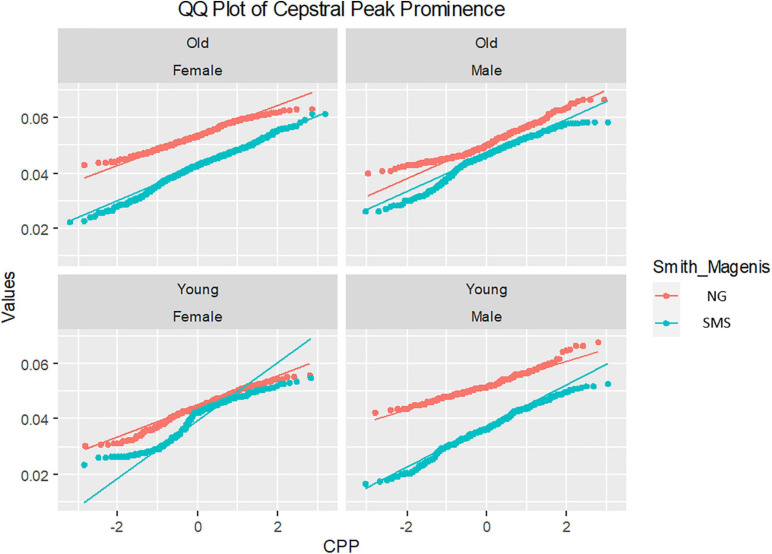
Q–Q plot cepstral peak prominence (CPP) distributions of gender and ages groups. Notice that young and old legend refers to ages of groups (ranges 1 and 2, respectively).

Likewise, the four Q–Q plots are depicted in Figure 8. After this second analysis, the Q–Q plot further highlights the differences between the two study groups (SMS and NG). The distributions are very different, especially in the group of young boys and older girls.

## Discussion

Our initial objective was to study the phonation and speech of children with SMS by means of an acoustic analysis to detect features associated with the neuromotor deficits that result from this genetic disorder. The main features of the neurological profile of patients with SMS can be summarized as: marked developmental delay, hypotonia, hyporeflexia, high pain threshold, poor motor skills, abnormal limb tremor, among others (Greenberg et al., 1996; Gropman et al., 1998, 2006, 2007; Wolters et al., 2009). It is possible that this picture of abnormalities is reflected in speech and phonation mechanisms, so at the beginning of this study, we expected to find different acoustic features in the speech emissions of patients with SMS compared to the normative population. For this purpose, a first observation of the articulatory particularities of SMS was made through the analysis of F1 and F2 of a sustained /a/ produced by 12 children with SMS between 5 and 12 years of age, who were compared with 12 age-matched children. As discussed in the previous section, the results of these analyses were not as expected, as the statistical tests did not yield relevant results, but we cannot come to any conclusions given the small number of participants. To attempt to arrive at robust results, it would be necessary to recruit more cases with SMS with a view to continue this research work. It would also be interesting to include tasks other than sustained /a/ to see if the results of the articulation analysis reflect the differences that were assumed at the beginning of this research.

After this first articulatory analysis, phonation was studied by determining the prominence of the cepstral peak. CPP values, unlike F1 and F2, were significantly different between the SMS group and NG. CPP is an acoustic parameter currently considered to be the most consistent parameter in the detection of dysphonia (Latoszek et al., 2018). It has been found to have a strong correlation between cepstral values and dysphonia severity. Consequently, it is also a widely used parameter in the analysis of dysarthria, as has been shown to be sensitive to phonation disorders of neurological origin. The outcomes of the phonation analysis indicated that there were significant differences between the SMS group and NG; it can be observed that in most of the cases with SMS, the CPP had a lower value than that in NG. This indicated that children with SMS had poorer vocal quality than their NG peers. CPP was less prominent in voices with reduced vocal quality, i.e., when their first harmonic did not stand out sufficiently from the background noise. The richer the harmonic structure of a voice, the higher the CPP; in other words, the better the quality of a voice, the more prominent the cepstral peak (Maryn et al., 2009, 2010; Lowell et al., 2011; Watts and Awan, 2011; Moers et al., 2012; Peterson et al., 2013; Samlan et al., 2013; Heman-Ackah et al., 2014; among others). The poor harmonic structure of a voice is linked to dysphonia, which can be organic, functional, and of a neurological nature (dysarthria). The latter are due to an alteration of the set of cortical and subcortical structures underlying the phonation mechanisms, whether degenerative, acquired, or developmental, as in the case of SMS. Thus, low CPP values have been evidenced in populations with Parkinson’s disease (Burk and Watts, 2018; Cushnie-Sparrow et al., 2018; Alharbi et al., 2019; Brown and Spencer, 2020; Novotný et al., 2020), ataxia (Jannetts and Lowit, 2014; Carson et al., 2016; Vogel et al., 2017), cerebral palsy (van Brenk and Kuschmann, 2018; Kuschmann and van Brenk, 2019), and even neurodevelopmental deficits, such as the Williams syndrome (Watts et al., 2008). The latter is, by far, the most similar clinical condition to SMS, as both are caused by a genetic abnormality and are conditioned by a neurodevelopmental disorder in the embryonic stages.

In the first study analysis involving single-case comparisons of phonation, the results clearly showed differences in laryngeal biomechanics for the children with SMS as compared with age and gender matched typically developing children. There were only a few cases in which no statistically significant differences had been found. However, if we take a closer look at some of these cases, we can see different distributions (Figures 4, 5). The Q–Q plot showing the distributions of SMS6 and 637A (Figure 5) showed that these two cases had different trends. The distributions intersected at a certain point, which could cause the test statistic to fail to distinguish the two samples as different and caused the *p* value to be above 0.05. In fact, the graph showed that the distributions separated. This phenomenon occurred similarly in the other cases where the statistical tests did not reject the null hypothesis.

In addition, what was noteworthy was that most of the cases assessed showed clearly different distributions (examples in Figures 6, 7). This finding would suggest that the altered neuromotor profile of SMS individuals influences the biomechanics of the structures involved in their phonation and voice quality. Apart from the neurological features described above, it has been shown that the population with SMS also has swallowing difficulties, hypotonia of the velopharyngeal and orofacial muscles (Sonies et al., 1997; Solomon et al., 2002), and alterations in the peripheral nervous system (75% of children with SMS) (Greenberg et al., 1996). Phonological difficulties and many simplification processes related to velopharyngeal hypotonia (distortions affecting velar and, mainly, fricative consonant phonemes) have also been described (Hidalgo and Garayzábal, 2019). Therefore, taking these findings into account, it would seem justified to relate the results of the acoustic analysis of this paper to the neuromotor particularities of SMS.

In the second study analysis involving subgroups of children divided by age and gender, children with SMS were clearly differentiated from their typically developing peers through the phonatory analysis of CPP. Power of statistical tests was enhanced by the large number of analyzed samples, as reflected in *p* values that were often well below 0.05 (e.g., young boys and old girls). Likewise, the Q–Q plots obtained generally presented separate distributions. As hypothesized, these results reinforce the fact that the use of the CPP as a phonatory feature helps to distinguish between healthy control and Smith–Magenis populations.

## Conclusion

This study is of an exploratory nature, but it has allowed us to identify how the phonatory characteristics of children with SMS differ with their typically five developing peers. These phonatory differences are likely associated with the neurological deficits that characterize of this syndrome.

An acoustic analysis of a sustained and comparative /a/ was carried out between an experimental group of 12 SMS cases aged 5–12 years and a control group of 12 typically developing children matched in age and gender. The initial aim was to determine whether the phonation and speech of the experimental group showed acoustic features that differed from the normative population. This interest stems from the altered neuromotor profile of the population with SMS and the close relationship between speech and phonation mechanisms and neurological disorders (dysarthria). Therefore, F1 and F2 were analyzed, as well as CPP, considered the most reliable acoustic parameter in the detection of dysphonia.

The main findings of this work can be summarized as follows. For the F1 and F2 analysis, no significant differences were found between the SMS children and the normative comparison group. Although this would not imply that both samples present with equivalent articulatory features, it is also possible that the F1 and F2 analysis for a single vowel was not sensitive enough to detect possible articulatory differences. In contrast, the phonatory cepstral analysis revealed significantly lower CPP for the children with SMS as compared with the age- and gender-matched normative group. Additionally, the vocal quality of most children with SMS in this study was lower than that of the normative comparison group. These findings suggest that the neuromotor deficits that characterize children with SMS may adversely affect laryngeal biomechanics and thus vocal quality. Finally, these findings are in line with previous research addressing dysarthria populations. The current results are also consistent with the findings made by Watts, Awan, and Marler in 2008 in a population with a syndrome with similar characteristics to SMS: Williams syndrome.

It is also important to point out some limitations of the present study. First, the experimental sample is small, although this is a frequent circumstance in research with rare and minority disease populations that are also underdiagnosed and relatively new. Second, another limitation has been the availability of a single speech task (a sustained /a/), since having several utterance tasks would have allowed us to test whether there are indeed no significant articulatory differences between SMS and typically developing children. In this sense, it will be interesting to examine the vowel quadrilateral for children with SMS and comparing that to a control group. In future research, authors will try to increase the population sample and the types of speech exercises to continue with the same line of study of the present work and to provide answers to unresolved questions. In addition, we will try to answer other questions that have arisen after this first analysis, such as the following: Is the typical neuromotor profile of SMS reflected in the same way in the phonation and speech of girls and boys? Is there a gender bias? Do adults with SMS present with a speech and voice profile that is further from normal than those diagnosed at a younger age? How do the speech and voice profiles of children with SMS compare to those of other syndromes with neurodevelopmental alterations? Our current, exploratory study addressed the initial purpose of determining whether acoustic features of speech and voice differed for children with SMS as compared with a normative group and confirmed that cepstral-based, phonatory features differed between the two groups.

## Data Availability Statement

The datasets presented in this article are not readily available because the law RGPD of Spain. Requests to access the datasets should be directed to DP-A, daniel.palacios@urjc.es.

## Ethics Statement

The studies involving human participants were reviewed and approved by ASME. The parents and/or legal representatives of each participant signed an informed consent accepting the participation in the process of data gathering, custody, and treatment. All data are kept in safe custody accordingly to European and national regulations. The Spanish Association of Children with Smith-Magenis Syndrome issued a letter to the name of the researchers acknowledging and availing the purpose and safety of the recording protocols. The protocols and procedures for data gathering and treatment adhered strictly to the Declaration of Helsinki.

## Author Contributions

IH-D, EG-H, PG-V, and DP-A contributed to the conception and design of the study. RM-O organized the database and extracted all features DP-A performed the statistical analysis and plots. IH-D, EG-H, RM-O, and DP-A wrote sections of the manuscript. All authors contributed to manuscript revision, read, and approved the submitted version.

## Conflict of Interest

The authors declare that the research was conducted in the absence of any commercial or financial relationships that could be construed as a potential conflict of interest.
